# Unilateral Urogenital Disontogeny in a Dog

**DOI:** 10.1155/2021/8831551

**Published:** 2021-04-13

**Authors:** Adolfo Maria Tambella, Stefano Martin, Matteo Cerquetella, Daniele Spaziante, Angela Palumbo Piccionello, Andrea Marchegiani, Umberto Faccenda, Giacomo Rossi

**Affiliations:** ^1^School of Biosciences and Veterinary Medicine, University of Camerino, via Circonvallazione, 93, 62024 Matelica, Italy; ^2^Futuravet Veterinary Clinic, Largo ‘815, 10, 62029 Tolentino, Italy

## Abstract

The purpose of this report was to describe an uncommon congenital anomaly in a dog. An 8-year-old, mixed-breed, male dog, was referred because of progressive difficulties on defecation. A complete diagnostic work-up (hematological analysis, radiology, ultrasound, and computed tomography), followed by surgery and histopathology, allowed us to diagnose the condition as unilateral urogenital disontogeny. The disorder was characterized by unilateral anomalies of the urinary tract (ectopic and dilated hydroureter, hydronephrosis, and renal dysplasia) associated with ipsilateral anomalies of the genital system (partial permanence of the duct of Wolff evolved into an epididymal-like structure and testicular agenesis). En bloc surgical excision of the complex of urogenital anomalies was performed with no complications during or after surgery. Surgery was considered to be effective in this dog since he no longer showed clinical signs of illness.

## 1. Introduction

Ectopic ureter is a well-known rather rare condition in the dog [1]; it is frequently associated with other abnormalities of the urinary tract, including dilated ureter, hydronephrosis, and renal aplasia-hypoplasia [2–8]. Genital abnormalities, associated with ectopic ureter, have been described both in females and in males even if with a low incidence [8, 9].

Although reports describing urogenital abnormalities are present in literature [3, 8–10], the present case is very peculiar as finding an unusual complex of congenital unilateral anomalies found in the urinary tract (ectopic hydroureter, hydronephrosis, and renal dysplasia) and genital system (partial permanence of the Wolffian duct, which evolved into an epididymal-like structure, and testicular agenesis) of an 8-year-old, male dog. The congenital anomaly remained asymptomatic up to this age, when an intra-abdominal mass effect caused by the bulky anomalous structures was probably emphasized by acquired prostatic hypertrophy.

## 2. Case Description

An 8-year-old, male, mixed-breed dog, weighing 22 kg, was evaluated at the Veterinary Teaching Hospital, School of Biosciences and Veterinary Medicine, University of Camerino, for progressive difficulties on defecation and tenesmus. The onset of the problem was uncertain, even if steadily worsening clinical signs were lasting from several months. During the two weeks' previous clinical evaluation, the dog also presented moderate dysorexia and depression. Clinically, the dog appeared reluctant to move and only one testicle was present in the scrotum. The abdomen appeared enlarged due to the presence of a nonpainful, fluctuating structure easily identified by abdominal palpation, and occupying most of the caudal and ventral abdominal cavity. By palpation, it was not possible to differentiate this structure from the urinary bladder. Digital rectal examination allowed evidencing an enlarged prostate with loss of normal lobes' separation. No other noteworthy clinical signs were found.

Complete blood count (white blood cells 6.73 × 10^3^/*μ*L, reference range 6‐12 × 10^3^/*μ*L; red blood cells 6.02 × 10^6^/*μ*L, reference range 6‐9 × 10^6^/*μ*L; platelets 236.0 × 10^3^/*μ*L, reference range 150‐500 × 10^3^/*μ*L), serum creatinine (1.17 mg/dL, reference range 0.5-1.5 mg/dL), blood urea nitrogen concentrations (BUN, 53.0 mg/dL, reference range 21.4-60.0 mg/dL), and other biochemical parameters showed no relevant abnormalities. Urinalysis, sampled by spontaneous urination, revealed the presence of leucocytes, erythrocytes, and numerous granular casts and sperm.

**Figure 1:**
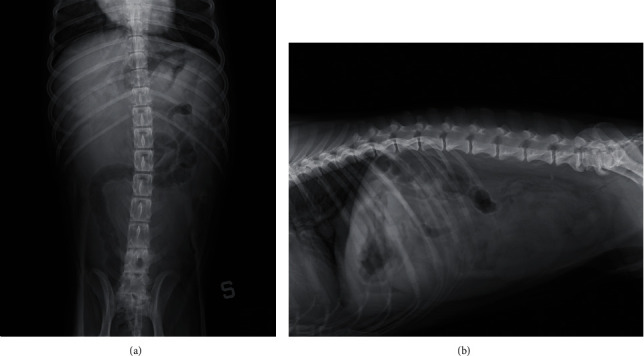
Abdominal plain radiographs: (a) ventrodorsal projection showing the large structure with a liquid-like radiopacity occupying a large part of the middle and caudal abdomen; right dislocation of the middle and distal portions of the descending colon (S indicates the left side); (b) right lateral projection showing the large and convolute structure localized cranially and dorsally to the urinary bladder; lumbosacral transitional vertebra and spondylosis in L6, L7, and S1.

Radiographs of the abdomen revealed, both in lateral and in ventrodorsal projections, a large and convolute soft tissue structure with a liquid-like radiopacity. It was localized cranially and dorsally to the urinary bladder, occupying a large part of the middle and caudal abdomen. The aspect and the opacity of that structure were similar to the urinary bladder, but it was clearly separated from it. In ventrodorsal projection, the mid and distal portions of the descending colon were evidently displaced from the natural position in the caudal left abdomen toward the right of the abdominal midline. A lumbosacral transitional vertebra was evidenced in a lateral view. Epiphyseal spurs were found at the caudal margins of the vertebral bodies in L6, L7, and S1. These signs of spondylosis were evident in both lateral and ventral views (Figure 1).

**Figure 2:**
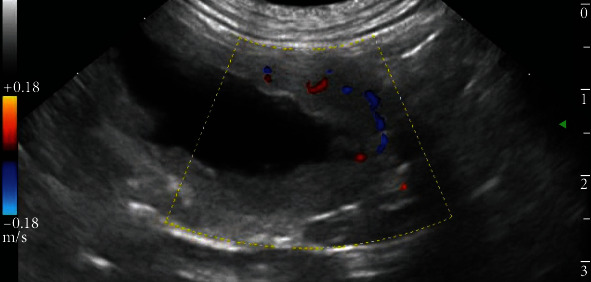
Abdominal ultrasound, left lateral approach. Cranial end of the fluid-filled tubular structure identified as the left hydronephrotic/atrophic kidney. Some tracts of renal vascularization are still evident.

Ultrasonography of the abdomen was also performed (MyLab™ Class C—Esaote, provided of a multifrequency microconvex probe 3.0-9.0 MHz). Middle portions of the abdomen were occupied by a fluid-filled tubular structure, endowed by an own wall; the structure was convoluted and folded on itself in some points. The fluid appeared mostly not corpusculated, but the one found in a caudal curvature presented suspended material. To the cranial end of the structure, positioned where the left kidney usually lies, a dead-end, whose wall was characterized by a thin vascularized parenchyma, it was present (Figure 2). The parenchyma appeared similar to the renal cortex and also the vascularization, where present, remembered anatomically the one of the kidney. The distal end of the tubular structure ended within the prostate that was enlarged and inhomogeneous, presenting diffuse anechoic vacuolations. The stomach, liver, spleen, bladder, right kidney, and right ureter were normal. No free cavitary fluid collection was found in the abdomen.

The structure described was assumed to be an ectopic and ectasic ureter terminating cranially in the remnant of the renal parenchyma of a markedly hydronephrotic left kidney and caudally in an enlarged and abnormal prostate. The left testicle was not detected by ultrasonography.

In order to better define the investigated structure and plan a possible surgery, a computed tomography (CT) of the abdomen was also performed (CT/E G.E., General Electrics Co.). The dog was placed under general anesthesia in dorsal recumbency. CT scans with 2 mm slice-width were performed craniocaudally, before and after intravenous infusion of water-soluble iodinated contrast medium (Iomeron 300 solution for injection, Bracco).

The CT did not show the left kidney in its normal location with respective failure to display the left renal artery and vein. The left adrenal gland was in eutopic location with a phrenic abdominal vein dividing the two poles. The right kidney, the right ureter, and the respective vascularization were normal for location and appearance.

The CT examination accurately demonstrated the characteristics of the well-defined tubular, convoluted, space-occupying, cystic structure, whose cranial border was at the bifurcation of the iliac artery and the caudal border in the pelvic area. The structure had liquid endoluminal content (12 Hu, Hounsfield units) and hyperdense wall (thickness of 0.2-0.3 cm) not enhancing contrast medium in venous and delayed phases. In the venous phase, CT images showed a slightly thickened portion of the wall (0.4 × 1.1 cm) located at the cranial dead-end of the saccular structure slightly enhancing the contrast medium. This portion was assumed to be the atrophic left kidney. In the delayed phase, the contrast medium was gathered in the lumen of the cranial portion of the saccular structure, very close to the atrophic kidney. The saccular structure was assumed to be the ureter that appeared considerably dilated (maximum width of 12 cm). The urinary bladder showed normal features and was surrounded by the saccular structure and slightly compressed by it from the left side.

The CT images demonstrated a hypertrophic prostate with a significant increase in prostate volume: the right lobe was 5.2 cm in diameter; the left lobe was 5.6 cm in diameter; the MPR (multiplanar reformat) reconstruction showed a dorsal diameter of 4.3 cm. A hypodense round shape area of about 1.7 × 1.4 cm at the level of the left lobe was recognized as a prostatic cyst. These CT findings were confirmed also by the histological examination of biopsies collected from the gland.

**Figure 3:**
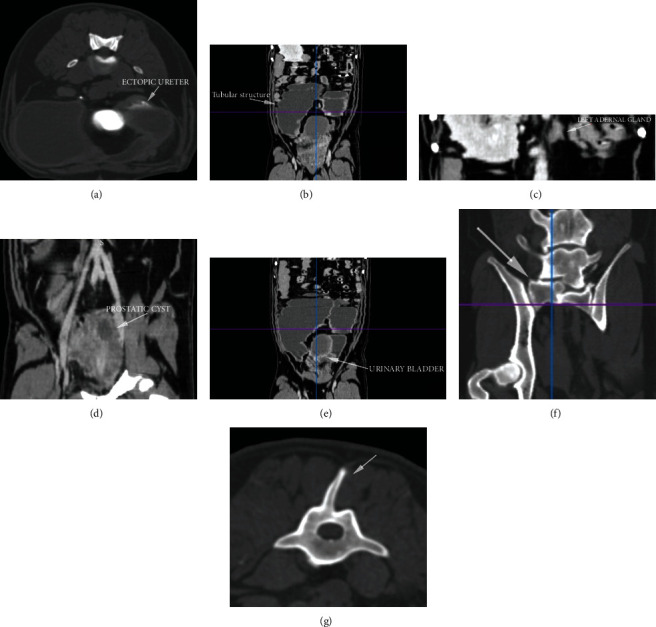
Abdominal computed tomography (CT): (a) transverse scan showing the ectopic megalic ureter; (b) multiplanar reformat (MPR) reconstruction showing the tubular structure; (c) MPR reconstruction showing the left adrenal gland; (d) MPR reconstruction showing the hypertrophic prostate with a prostatic cyst; (e) MPR reconstruction showing the urinary bladder surrounded by the convolute saccular structure; (f) MPR reconstruction showing the lumbosacral transitional vertebra; (g) transverse scan showing the 4^th^ lumbar vertebra with left lateral deviation of the dorsal spinal process.

CT images also defined an anomaly of the spinous process of the fourth lumbar vertebra with its lateral deviation to the left side. CT images confirmed the presence of lumbosacral transitional vertebra and spondylosis in L6 and L7 and in the sacrum, already highlighted with plain radiographs (Figure 3).

The dog was moved to surgery, placed in dorsal recumbency, and maintained in general anesthesia to perform a ventral midline celiotomy. General anesthesia was managed for diagnostic imaging and surgery with intravenous injection of 3 *μ*g/kg dexmedetomidine (Dexdomitor; Orion Pharma, Italy) and 0.2 mg/kg methadone (Semfortan; Dechra, Italy) mixed in the same syringe. Ten minutes after the premedication, anesthesia was induced with intravenous injection of 2 mg/kg propofol (Propovet; Vetfol, Italy). Following orotracheal intubation, anesthesia was maintained with isoflurane (IsoFlo; Zoetis, Italy) in 100% oxygen, for performing CT and surgery. During surgery, the dog received a loading dose of 2 mg/kg lidocaine (lidocaine 2%; Ati, Italy) followed by a constant rate infusion of 100 *μ*g/kg/minute.

A ventral midline celiotomy allowing a comprehensive exploration of abdominal organs was then performed. On opening the abdomen, it was immediately highlighted a bulky, fluctuating, convoluted, bag-shaped structure, occupying most of the caudal region of the abdomen. During the exploration of the abdominal cavity, no noteworthy changes in the liver, spleen, pancreas, gastrointestinal system, right kidney, and bladder were found, while the left kidney appeared absent.

A more thorough exploration of the saccular structure revealed that it was not in a direct anatomical relationship with the bladder, as it was clearly extraperitoneal. At the direct palpation, it was appreciated an obvious, generalized fluctuation for abundant collection of fluid within; nevertheless, just at its dorsal-cranial extremity, it was possible to appreciate a small area (whose maximum diameter was about 1.5 cm) with a more firm and elastic consistence and darker in colour than the surrounding areas, attributable to a residue of the left kidney covered by parietal peritoneum. The complex saccular structure was identified as markedly dilated ureter and renal pelvis. In parallel and ventrolaterally to it, a white tubular structure, accompanied by a small vascular trunk, was identified as a sketch of the genital annexes and the *vas deferens*.

The connection between the megaureter and the lower urinary tract occurred at the level of the prostatic urethra, jointly to the *vas deferens*, thus bypassing completely the bladder trigone, allowing a further diagnose of extramural ectopic ureter.

**Figure 4:**
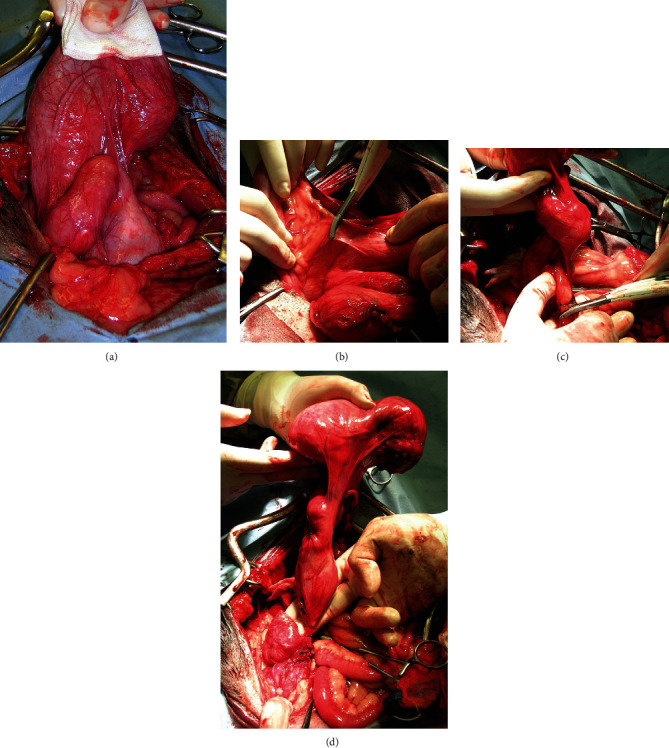
Stages of surgery: (a) ventral midline celiotomy and exploration of part of the large, fluctuating, convolute structure occupying a large portion of the abdomen; (b) dissection of peritoneal anchors to isolate the complex of megaureter, renal pelvis, residue of the kidney, *vas deferens*, and sketch of the genital annexes; (c) sealing of the vascular supply; (d) ligatures of the distal portion of the ectopic ureter and *vas deferens* in proximity to the prostate gland to fulfill the nephroureterectomy and deferentectomy.

**Figure 5:**
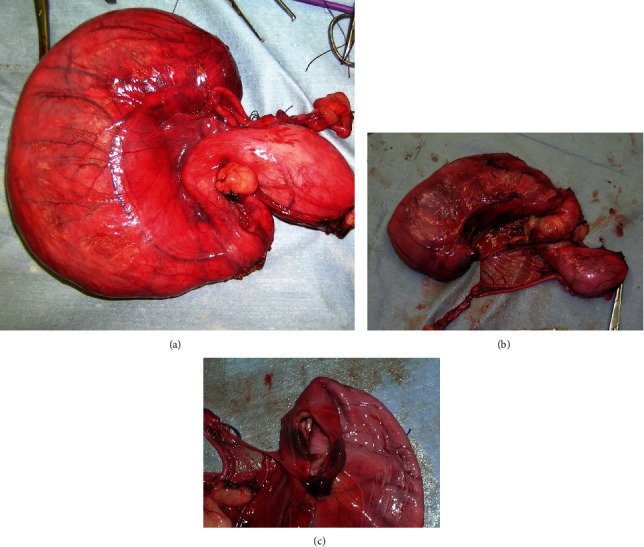
Complex of megaureter, renal pelvis, dysplastic kidney, *vas deferens*, and sketch of the genital annexes after excision: (a) ventral aspect; (b) dorsal aspect; (c) detail of the dysplastic-small kidney after sectioning and emptying the megaureter.

The complex of megaureter, renal pelvis, residue of the kidney, *vas deferens*, and sketch of the genital annexes was isolated through blunt and sharp dissection. An electrothermal bipolar vessel-sealing device (LigaSure Small Jaw™ handpiece connected to a ForceTriad™ Energy Platform, Valleylab Covidien) contributed to reduce the operating time and intraoperative bleeding during the dissection of the parietal peritoneum removing en bloc the entire structure. A double ligature and subsequent cutting of the distal portion of the ureter and *vas deferens* were performed closeness to the prostate gland to conclude the nephroureterectomy and deferentectomy (Figures 4 and 5). A biopsy of the prostate was performed by a true-cut needle. The abdominal wound was closed in routine fashion. The eutopic right testicle was removed by prescrotal open orchiectomy.

**Figure 6:**
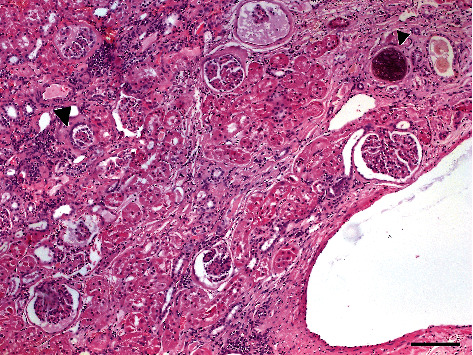
Histological aspect of renal parenchyma of 8-year-old mixed-breed dog with ectopic ureter: note the characteristic dysplastic aspect of the kidney, with loss of corticomedullary differentiation, containing some well-developed glomeruli, admixed to small cysts and primordial glomeruli (big arrow) surrounded with a cubic-to-cylindrical epithelium. Some glomeruli are calcified (small arrow). H&E, scale bar = 300 *μ*m.

Histological evaluations performed on removed tissues confirmed the presence of congenital ectopic hydroureter associated with hydronephrosis and renal atrofia, associated with unilateral genital disontogeny, partial permanence of the duct of Wolff evolved into an epididymal-like structure, and testicular agenesis. In particular, histology of the affected kidney revealed a typical dysplastic organ, composed in part, of undifferentiated and metaplastic tissues belonging to metanephros, that represents the direct precursor of the mature kidney, and consisting in various ureteric bud branches from the mesonephric duct, with renal mesenchyme condenses around the advancing bud-forming nephrons. This dysplastic-small kidney (ranging in size between 3.5 and 4 cm) has loss of corticomedullary differentiation, containing some well-developed glomeruli, admixed in the peripheral portion of the parenchyma to small cysts surrounded with a cubic-to-cylindrical mono- or bilayered epithelium (Figure 6).

**Figure 7:**
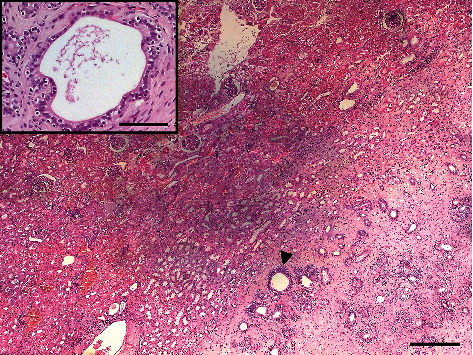
Histological aspect of the embryonal parenchyma of the left kidney: note that in the central portion of parenchyma, a well-developed Wolffian duct residue (arrow) is evolving, in the distal portion, into an epididymal-like structure. Insert: a Wolffian duct residue with a columnar epithelium and a lumen patent along its whole length is observed. H&E, scale bar = 600 *μ*m. Insert, scale bar = 150 *μ*m.

Distally, a well-developed Wolffian duct with a columnar epithelium and a lumen patent along its whole length was observed, evolving into an epididymal-like structure, composed of dilated and azoospermic tubuli lining by typical epithelium, and surrounded by a poorly developed venous pampiniform plexus. Total testicular agenesis was histologically confirmed (Figure 7).

The dog was evaluated for the last time 18 months after the surgery, and the clinical condition was good and stable; no abnormalities at defecation and urination were found. Urinalysis did not show any alteration. Complete blood count (white blood cells 10.0 × 10^3^/*μ*L, reference range 6‐12 × 10^3^/*μ*L; red blood cells 6.28 × 10^6^/*μ*L, reference range 6‐9 × 10^6^/*μ*L; platelets 339.0 × 10^3^/*μ*L, reference range 150‐500 × 10^3^/*μ*L), serum creatinine (1.09 mg/dL, reference range 0.5-1.5 mg/dL), blood urea nitrogen concentrations (BUN, 34.7 mg/dL, reference range 21.4-60.0 mg/dL), and other biochemical parameters were within normal ranges.

## 3. Discussion

Hormonal disorders during embryonic development could influence the normal growth of male gonads and Wolffian ducts, keeping the Mullerian duct, that could potentially develop in a uterus-like structure [11, 12]. In males, the persistence of the Mullerian duct is well described [13–15]. The anatomical alterations of the urogenital system are quite similar to the case reported herein. In PMDS (persistence of the Mullerian duct syndrome), it is possible to observe a hypoplasia/agenesis of testicles, anomalies of deferens ducts, and urinary tract anomalies [13–15]. The typing of the karyotype of the affected patient suggests a genetic and hereditary predisposition [13, 15]. In female patients, the association of hypoplasia/agenesis of the kidney and ovary and the presence of ectopic ureter or other urogenital tract alteration are observed in subjects affected by Gartner cyst or Gartner duct [16–22]. In such patients, the normal embryonal regression of the Wolffian duct did not occur completely, demonstrating several degrees of severity [20], and they could have various degrees of communication between Gartner formation and urogenital system [17].

The peculiarity of this clinical case reported was represented by the persistence of the Wolffian duct in an adult male patient. In light of this, it was not possible to catalogue findings of this case as Gartner cyst or duct, although the same structures are partially involved. Another characteristic element of our case was represented by severe alteration of the ipsilateral kidney and ureter. In particular, a “small kidney” was observed.

Histopathologic examination is used to distinguish various etiologies among small kidneys, because this distinction is important for disease prognosis and genetic counseling. The diagnosis of renal dysplasia is not difficult; however, the diagnosis is sometimes confused with other conditions including polycystic kidney disease, fetal kidney, renal hypoplasia, and renal atrophy. The dysplastic kidney contains primitive ducts with or without dilated cysts [23]. The fetal kidney contains poorly differentiated tissues that are compatible with gestational development. The hypoplastic kidney has fewer nephrons than the normal kidney, but no dysplastic elements [24] as observed in our case. The atrophic kidney exhibits segmental loss of parenchyma due to renal scarring and compensatory hypertrophy in the remnant parenchyma. The metanephros is the direct precursor of the mature kidney, and perturbation of mesonephric duct differentiation, during the advancing bud and formation of nephrons and ureter and collecting ducts, underlies the spectrum of disorders called congenital anomalies of the kidney and genitourinary tract [25]. Only histologically, a correct diagnosis between the two categories: renal dysplasia or hypoplasia, is possible. In our case, a partial permanence of the Wolffian duct, which is evolved into an epididymal-like structure, and testicular agenesis was also documented.

It is noteworthy that a dog presenting an ectopic ureter and other urogenital abnormalities was not referred for urinary incontinence but rather for difficulties on defecation. Probably, the joint pathogenetic action deriving from congenital urogenital anomalies and benign prostatic hyperplasia induced a mass effect on the surrounding abdominal organs and triggered the clinical manifestation.

Many authors [3, 4, 6, 8, 26–29] reported that in females, urinary incontinence is a frequent symptom in case of ectopic ureter; probably, in the case herein described, urinary incontinence did not occur because it has been hypothesized that in males, the higher length of the urethra and the higher intraluminal pressure in prostatic urethra could prevent the development of incontinence [3, 8, 28, 29].

It is likely that the worsening of the general conditions of the last weeks, characterized by dysorexia and depression, could be related to renal and ureteral conditions both getting worse because of the enlarged prostate probably progressively partially compressing the ureter. Probably, the volumetric bulk of the ureter may have contributed to the development of symptoms by exerting a mass effect on other abdominal organs.

Another interesting aspect of this report is the age of dog (8 years) at the diagnosis of the urinary condition, being that the average age of diagnosis of ectopic ureter is lower [2, 3, 5, 29]. As previously reported in dogs presenting ectopic ureters [29], also in this case, no clinically relevant hematologic anomalies were found; in the author's opinion, urinalysis was compatible with the clinicopathological condition found, especially being that the urine sample was collected by spontaneous urination and not by cystocentesis.

Surgical correction is considered the main treatment for ectopic ureter: the choice of surgical technique is based on the characteristics and extent of the alterations, such as the number, morphology and functionality of ectopic ureters, the location and appearance of the ureteral outlet, the functionality of the kidney, and the presence of other concomitant malformations. The neoureterostomy is generally performed in case of intramural ectopic ureter, while the ureteroneocystostomy is the preferred technique in case of extramural ectopic ureter [8, 9, 30–32]. The indication for the nephroureterectomy is restricted to ectopic ureters with unilateral, nonfunctional, and irreversible alterations of the ipsilateral kidney [1, 30–32]. The serious alterations observed in this case have led the authors to perform a nephroureterectomy. The surgical technique allowed to complete the surgery with no complications during or after surgery and resulted to be an effective therapy for the problem of dog.

In this report, an unusual complex of urogenital disontogeny was described in an adult male dog. The presence of a congenital disease should be always included in the differential list also in adult patients, and a complete diagnostic work-up should always be performed as the apparent anatomical location of clinical signs may not match the real location of the problem. Surgery confirms to be the resolutive approach in most of anatomical malformations.

## Data Availability

All data are included in the main text of the manuscript.
